# Polyclonal IgG therapy in neurologic and neuromuscular disease: mechanisms of action and insights from newer therapies

**DOI:** 10.3389/fneur.2026.1819474

**Published:** 2026-10-09

**Authors:** Mel Berger

**Affiliations:** Departments of Pediatrics and Pathology, Case Western Reserve University, Cleveland, OH, United States

**Keywords:** autoimmunity, CIDP, GBS, IgG therapy, IVIg, MMN, myasthenia gravis, neuropathy

## Abstract

Plasma-derived polyclonal IgG (IVIG or SCIG) has become the mainstay of therapy for autoimmune peripheral neuropathies including Guillain-Barre Syndrome, Chronic Inflammatory Demyelinating Polyradiculopathy (CIDP) and Multifocal Motor Neuropathy, as well as Myasthenia Gravis. Use in these diseases exceeds that for all immune deficiencies combined. Laboratory and animal studies suggest many diverse mechanisms of action of IgG on cellular and humoral immunity. However, the spectrum of immune-mediated diseases in which IgG is efficacious is largely limited to conditions mediated by autoantibodies and/or complement. Careful analysis of clinical results indicates that only a few mechanisms are physiologically relevant: anti-idiotype neutralization, accelerated catabolism of IgG autoantibodies, and blockade of complement activation and deposition. These MOAs likely operate simultaneously and may contribute synergistically to the overall efficacy of IgG. Importantly, IgG does not act as a disease modifying drug, and repeat dosing at regular intervals is necessary in chronic diseases. This review seeks to provide a firm basis for understanding the mechanisms of action *in vivo*, and to provide benchmarks for understanding the effects and uses of new agents that have been recently approved and/or are under development.

## 1 Introduction

Polyclonal IgG contains a diverse population of IgG antibodies with many different specificities, as opposed to monoclonal products which are composed of identical antibody molecules with a single antigenic specificity. Plasma-derived polyclonal human IgG is widely used for autoimmune and inflammatory diseases in addition to its traditional role in antibody replacement for immunodeficient individuals. Global consumption of polyclonal IgG has been increasing by more than 6% per year and exceeded 300 metric tons in 2023 (1), requiring collection and fractionation of more than 60 million liters of plasma annually (2). Although defense against infectious disease is generally regarded as the major physiologic role of endogenous IgG, plasma-derived pooled polyclonal IgG has become a critical treatment for autoimmune diseases, especially those involving the neuromuscular system. The UK national health service database estimates that IgG use for chronic inflammatory demyelinating polyneuropathy (CIDP) alone is 88% of that for all primary immune deficiencies combined, and that utilization for chronic neuropathies exceeds its use for primary immune deficiencies by more than 35% (3). The marketing research bureau estimates that CIDP, gMG, MMN, and GBS account for 55% of all IgG use in the US (MRB 2024, Figure 1). As newer therapies with more specific single mechanisms of action are becoming available, careful analysis of clinical data on those diseases in which IgG is most effective and the actions which are most important in its overall effects should help us better understand and optimize the use of this valuable tool in our armamentarium.

**Figure 1:**
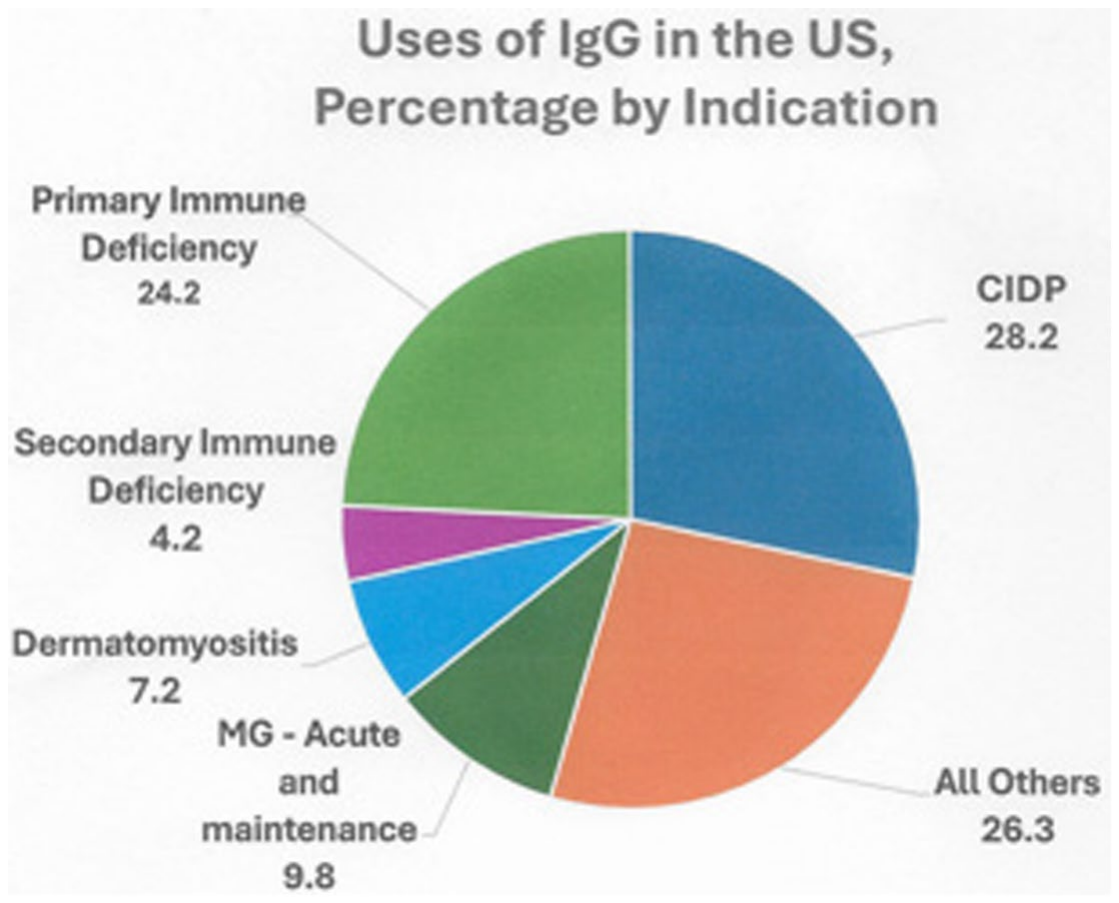
Usage of IgG in the US by Indication: Data from US (MRB). Data from the UK MDSAS are similar (in millions of grams): PID 1.4, SID 0.97, SAD 0.08, other Immune Def 0.018, CIDP 1.26, MMN 0.67, MG 0.73, GBS 0.014, other 0.039, Infectious dis 1.03, Hematology 0.31, Other 0.12.

### 1.1 Methods and limitations of this review

A PubMed search with the terms “Immunoglobulin,” “Intravenous” and “Mechanisms” returned 4,391 citations. Given the diversity of hypotheses, laboratory approaches and animal models employed, systematic reviews or meta-analyses of this type of data are not possible. This review is based on publications selected from PubMed and clinicaltrials.gov as well as FDA websites. In discussing clinical trials and results, emphasis has been on Cochrane and other systemic reviews and meta-analyses, particularly when comparing modalities with each other, like plasma exchange vs. polyclonal IgG and IgG vs. newer modalities. Wherever available, task force reports, professional society guidelines and regulatory agency approvals have been included.

The major effort in this review has been to reconcile clinical observations and controlled human studies with results of laboratory and animal experiments. Many diseases for which IgG is used are incompletely understood and the laboratory models are carefully constructed to single-out individual hypotheses. Thus, their relationships to each other are often difficult to discern. In those cases, preference has been given to the interpretation of clinical results. Unfortunately, dosing, pharmacokinetic and pharmacodynamic differences, and choices of enrollment criteria and endpoints confound direct comparisons between different treatments. Nevertheless, I hope this review proves helpful in discerning how the new therapies with clearer, single mechanisms of action relate to the overall effects of polyclonal IgG. The overall goal is to provide a framework for integrating results of studies of emerging therapies and to better understand the uses and limitations of polyclonal IgG and how and when it can be best employed now and in the future.

### 1.2 Historical perspective

Concentrates of plasma-derived IgG first became available as by-products of the industrial scale production of albumin in World War II, and were initially used for prevention of infectious diseases such as hepatitis A, measles, and polio (4, 5). In recent decades, vaccines have obviated much of the use of polyclonal IgG for prophylaxis in otherwise healthy individuals and travelers. Agammaglobulinemia was first described in 1952, and replacement therapy was initially given subcutaneously using 16% immune serum globulin (6), but higher doses (25 mg/kg/week) given intramuscularly became standard therapy. These injections were painful and poorly tolerated (5, 7). Shock-like reactions and other adverse effects precluded IV administration of IgG until the advent of low pH incubation and use of sugars or amino acids as excipients led to safer and better-tolerated IV preparations in the 1980s (4, 5, 8).

Shortly after the introduction of intravenous immunoglobulin (IVIG), serendipitous observations showed that high doses rapidly raised platelet counts in children and adults with immune thrombocytopenic purpura (ITP) (9–11). This was also effective in autoimmune neutropenia (12) and it neutralized autoantibodies to FVIII (13). This led to trials of high-dose IVIG in autoantibody-mediated neuromuscular diseases, including MG (14–16), GBS (17–19) and CIDP (20–22). Recognition of the efficacy of IVIG in MMN came later and it remains the only approved therapy for MMN (23, 24), Joint EFNS/PNS Task Force 2010 (25, 26).

IVIG and subcutaneous IgG (SCIG) have also become the mainstays of therapy for other chronic autoantibody mediated neuromuscular diseases including dermaytomyositis, stiff-person syndrome and others (27, 28). IVIG is also first line therapy for GBS. Because of the prevalence of CIDP, MMN, MG, and the need for long-term treatment with high doses at monthly or shorter intervals, neuromuscular diseases have become the major uses of polyclonal IgG, even though the mechanisms of action have not been fully delineated. Polyclonal IgG is widely used for other autoimmune and inflammatory diseases (29), but different MOAs are likely important in Kawasaki Syndrome and ITP (30, 31).

## 2 Preparation and characteristics of polyclonal human IgG products

The US FDA currently lists 12 approved polyclonal products for IV administration (IVIG) and 7 for subcutaneous administration.^1^ All are prepared from pools of thousands of units of plasma obtained under strict standards at FDA approved centers in the US, using variations of the Cohn-Oncley cold ethanol precipitation method developed during World War II. Some products use caprylate (octanoic acid) in place of one of the alcohol steps. All products undergo a pH 4 incubation and contain stabilizers such as the sugars maltose or sorbitol, or amino acids such as glycine or proline (8). All contain >95% IgG, with subclass compositions similar to normal plasma, but the IgA content of different products varies [see prescribing information for each product, or table on pp. 24–25 in the Immune Deficiency Foundation Product Booklet (32)]. All preparations include dedicated viral removal/inactivation procedures and no current products have transmitted any known blood-borne pathogen. Information on titers of specific antibodies and contents of different glycosylation isoforms are not publicly available and do not influence the FDA-approved indications for different products in the US.

Most IgG molecules bear N-linked glycans attached to Asparagine 297 on the Fc region and some also bear glycan side chains on Fab region. These glycans are highly heterogeneous, including variations in content and position of fucose, galactose, sialic acid, and N-acetylglucosamine, which can modulate the antibody’s structure and function (33). Some investigators reported that a small fraction of hyper-sialylated IgG molecules in IVIG preparations accounts for the majority of its anti-inflammatory activity by binding to the DC-SIGN receptor, which then alters FcR effector functions (34). However, this mechanism was observed mainly in particular animal models, and applications in human therapy have not been demonstrated. Different glycoforms of IgG have been shown to differ in efficacy in blocking complement deposition induced by GBS, MFS and MMN sera onto ganglioside-coated microtiter plates (35), but these results may be influenced by the interactions of sugar side chains on the IgG molecules with the antigen binding sites on the ganglioside, which also contain sialic acid. Different glycoforms have not been moved forward into therapeutic trials. Analysis by mass spectroscopy of 154 lots of 7 different IVIG preparations showed less than 3% variation in the content of different glycoforms in all the products tested (36). Gallia et al. (37) performed cross-over studies with 4 different IVIG products over 49 months in 6 patients with CIDP and 7 with MMN, and found no difference in disability scores with the different brands. Kuitwaard et al. (38) performed a randomized trial of two different preparations of IVIG in 27 CIDP patients and found no difference between the products in disability scores or strength measurements.

FDA-approved indications for polyclonal IgG products in the US include only immune deficiency, ITP, CIDP, MMN and dermatomyositis, but any individual product is approved only for those indications for which efficacy data has been accepted by the FDA. Interestingly, although IVIG is considered first-line therapy for Kawasaki Disease and GBS, those conditions have not received FDA approval, most likely because suitable placebo-controlled studies have not been submitted. (The one exception is that Gammagard S/D is FDA-approved for Kawasaki disease). In Europe, one core SmPC applies to all intravenous immunoglobulins (39) and includes the indications: replacement therapy for primary and secondary immune deficiencies (with serum IgG < 4 G/L or proven antibody insufficiency); for “immunomodulation” in ITP, Guillain Barré syndrome (GBS), Kawasaki disease, CIDP and for multifocal motor neuropathy (MMN).

A 2023 consensus statement from the American Association of Neuromuscular & Electrodiagnostic Medicine (AANEM) recommended IVIG for the treatment of chronic inflammatory demyelinating polyneuropathy, Guillain-Barré Syndrome (GBS) in adults, MMN, dermatomyositis, stiff-person syndrome and myasthenia gravis exacerbations (but not stable disease) on the basis of class I evidence (40). Similarly, EAN/PNS task forces “strongly recommended” IVIG as a first line treatment for CIDP (41) and for GBS (42) [reviewed by Allen (25)]. Full discussions of the data on efficacy and safety of IVIG as compared with placebo, plasma exchange and corticosteroids are beyond the scope of this review, but are available in the task force reports cited and various Cochrane Reviews (43–45).

## 3 Clinical use of polyclonal IgG

### 3.1 Kinetics and route of administration

In actual clinical practice in CIDP, MMN and other neuromuscular diseases, IgG doses and intervals vary widely, but are usually in the range of 1–2 g/kg every 2–4 weeks (46). Peak serum concentrations of IVIG are achieved as soon as the infusion is concluded, and may reach 3,000–5,000 mg/dL with high dose therapy (Clinical effects closely parallel the changes in IgG levels, Figure 2). Over the subsequent 2–3 days the serum IgG level falls by about half as IgG is distributed into the total extracellular fluid volume (47, 48). Following this equilibration, IgG is catabolized with first-order (concentration-dependent) kinetics: the half-life may be as short as 10 days after high-dose infusions (48), but is conventionally described as 21–28 days at normal IgG levels or following doses of 300–500 mg/kg in immunodeficient patients (Figure 3).

#### 3.1.1 Subcutaneous IgG (SCIG)

Subcutaneously administered IgG is deposited into the subcutaneous tissue then absorbed into and transported through lymphatics, entering the bloodstream at about the same rate as equilibration of IVIG out of the intravascular compartment, peaking in about 3–5 days (47). By eliminating the high peaks which follow large IV infusions and administering fractional doses at more frequent intervals, true steady state serum IgG concentrations can be maintained with SCIG, because the next dose is given before the trough bottoms-out (47, 49). Although the steady serum IgG level will be higher if the monthly IVIG dose is fractionated into 4 SCIG doses at weekly intervals, the overall area under the curve of concentration vs. time (AUC) is about 30% lower with SCIG, so an increment in the total monthly dose is often recommended, and was required by the FDA in licensing trials of SCIG. However, it should be noted that for some indications, such as GBS (50) and Kawasaki Syndrome (51) the height of the peak seems to be the most important pharmacokinetic parameter, while for prevention of infection, the trough level may be most important (52, 53). Thus, individual patient’s clinical responses should be carefully considered in adjusting maintenance doses and regimens. In general, SCIG is associated with fewer systemic adverse effects- eliminating the peaks in from high dose IV infusions seems to obviate many of the problems with headaches and rate-related infusion reactions, but SCIG is associated with local site reactions (54–56). IVIG is generally administered by a medical professional and monitored in a clinical setting or by a home-infusion nurse. In contrast, SCIG is generally self-administered at home.

Another route of IgG administration, termed facilitated subcutaneous IgG (fSCIG), utilizes a preliminary injection of recombinant human hyaluronidase (rHuPH20), which transiently depolymerizes hyaluronan in the extracellular matrix, temporarily increasing the permeability of subcutaneous tissue. The hyaluronidase pretreatment enables the administration of large infusion volumes with high infusion rates so that for equivalent monthly doses, less frequent infusions and fewer infusion sites are required. The hyaluronidase pre-treatment also improves the bioavailability of SCIG, so that the increment from IVIG does not need to be as great. Kinetic studies of fSCIG show that the rate of systemic absorption is about the same as for conventional SCIG, with 3–5 days required for reaching the maximum serum concentration (57). Also like conventional SCIG, the peak is truncated as compared to the peak achieved with IV infusions. A combination product containing separate vials of hyaluronidase and 10% IgG is marketed in the US as “HyQvia®” (facilitated subcutaneous IgG, fSCIG) by Takeda (Cambridge, MA) and is licensed for immune deficiencies and CIDP. In the major placebo-controlled licensing trial for CIDP, 132 subjects were included, with 62 randomized to receive HyQvia®. The fSCIG was administered at a mean monthly dose of 1.1 gr/kg, for a mean total of 85.4 gr. Eighty-eight % of the patients employed 2 sites at 4-week intervals, consistent with a volume of 420 mL/site, and the infusions required a mean (± SD) of 126 ± 49 min. Overall efficacy, as indicated by relapse rate as compared to placebo, was similar to other placebo-controlled studies of IgG in CIDP, but 37% of subjects showed functional worsening as indicated by decreases in INCAT or I-RODS scores or hand grip strength, as compared to their scores on previous IVIG therapy (58). Infusions were generally well-tolerated and adverse events were mostly mild to moderate local infusion site reactions. Eleven % of fSCIG subjects developed detectable antibodies against the hyaluronidase, but these were not neutralizing and were not associated with adverse effects (58). At the conclusion of the 6 month study, 67% of the fSCIG treated subjects reported a preference for remaining on the study drug, while only 11% preferred to return to their previous IVIG regimen (58). The dose of hyaluronidase is 0.5 mL per 10 mL of IgG. Both are administered through the same 24 ga needle, with the IgG infusion beginning 10 min after the hyaluronidase using a high-rate (up to 300 mL/h) infusion pump. If desired, HyQvia® may be self-administered at home after the patient has been adequately trained (Prescribing Information, Takeda Ltd).

Most currently available IV preparations generally contain 100 mg IgG/ml, equal to 10 g/100 mL (10% w/v) and are typically started at rates of 1–2 mg/kg/min and ramped up step-wise to 8 mg/kg/min as tolerated. Subcutaneous preparations may be as highly concentrated as 200 mg/mL (20 gr/100 mL = 20% w/v), and are typically given into multiple subcutaneous sites at rates of 20–30 mL/site/h and volumes ≤ 50–60 mL/site. Thus, IV infusions of 1–2 gr/kg (1000–200 mg/kg) generally require 3–4 h, or more if the highest tolerated infusion rate is less than 8 mg/kg/min. For SCIG, 50 mL of 20% solution contains 10 gr of IgG, so an 80 kg patient will require 8–16 sites per month, or 2–4 per week. Patients might use 2–4 sites simultaneously, so the monthly dose could be achieved with one or two infusions per week each lasting 1–2 h. Of course, with SCIG these could be given at the patient’s convenience in the comfort of their own home, while watching TV, reading or doing light activity. IVIG infusions are generally given using 16–18 ga needles in adults, while 24–26 ga needles are used for SCIG (59).

Case reports and cross-over studies suggest that SCIG and IVIG are equally effective in controlling symptoms and improving strength in CIDP and MMN patients and that the majority of patients switched from IVIG to SCIG choose to continue on SCIG, although these are not necessarily well- randomized studies, so selection bias cannot be ruled out (60–62). Similar or slightly higher total monthly doses of IgG are often required for optimal management with SCIG as compared to IVIG. A meta-analysis of a systematic review which included 9 studies with 110 MMN patients found no difference in improvements in strength or reduction of disability with SCIG as compared to IVIG (63). Most of these studies report that SCIG decreased treatment-related fluctuations in strength and disability (see example in Figure 2) and maintained or improved overall quality of life. A recent systematic review of quality of life studies in MMN and CIDP showed comparable results for the two modalities, with potential benefits of SCIG related to self-administration and decreased systemic adverse effects (64).

**Figure 2:**
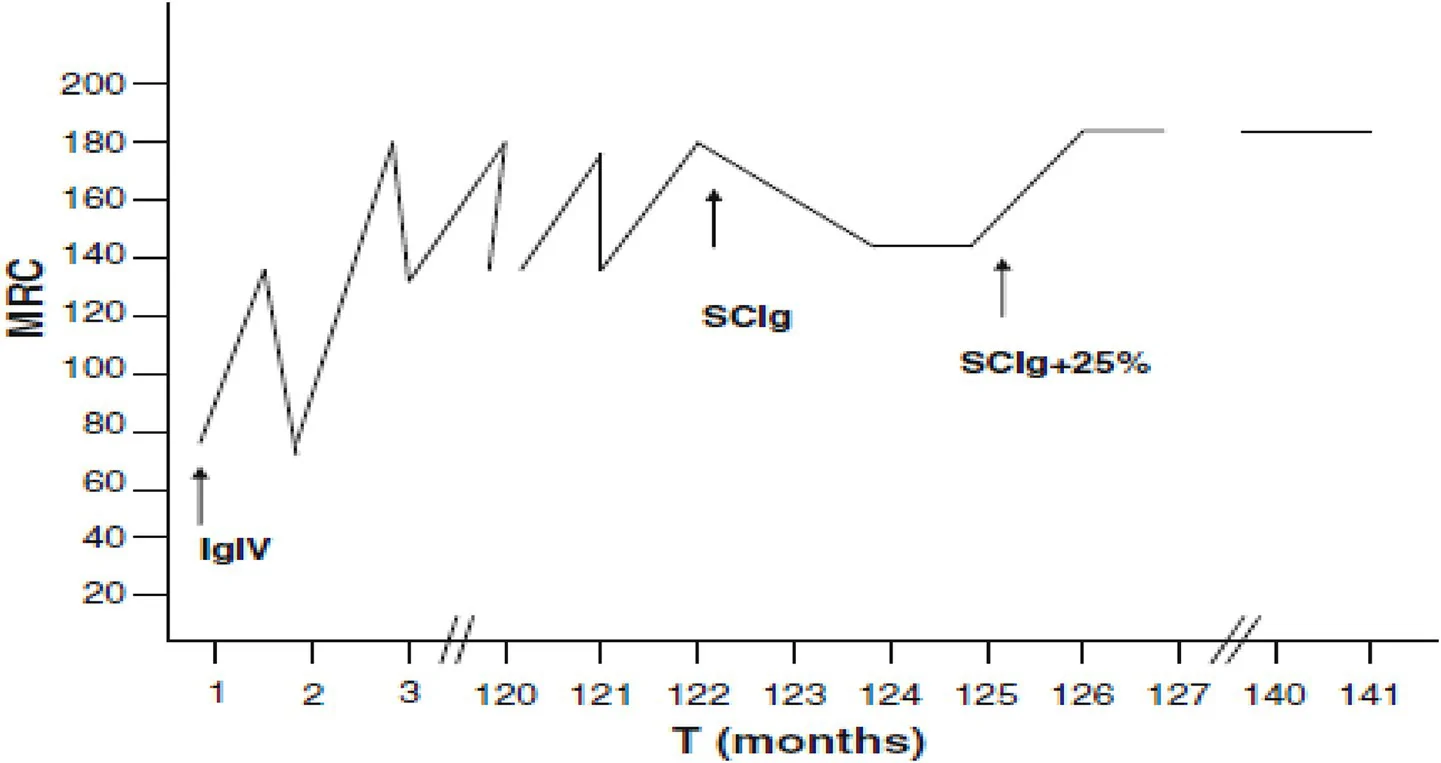
Response of an MMN patient to IV and SC IgG treatment (from: Dacci et al. (121)). y-axis: MRC muscle strength sum score. Initially, the patient was treated with 1 gr/kg of IVIG. Response to each infusion is indicated by the increase in MRC score. He had an end of dose effect every 3 weeks consisting of progressive weakness with inability to walk without support. He was then treated with SCIg at a monthly dose of 1 g/kg (equivalent to monthly IVIG dose) subdivided into four weekly doses. He initially worsened, corresponding to an IgG level of 1500 mg/dl, and required a dose increase of 25% of the total monthly dose. After this dose adjustment, patient’s strength remained stable under SCIG treatment with a stable serum IgG level of 2100 mg/dl. Reproduced with permission of the publisher.

For chronic use in CIDP, MMN or MG, dosing and intervals should be individualized based on each patient’s response, tolerance and preferences (54, 65, 66). Considerations in the choice of IVIG vs. SCIG regimens include: overall efficacy, treatment-related fluctuations in strength/disability, frequency and tolerance of systemic vs. local adverse effects, independence and self-reliance vs. preference for regular professional support and monitoring, ease of IV access vs. need for a “port” or other device, number of needle sticks (sites) and infusion location/travel. Cost comparisons for IV vs. SC IgG are difficult because monthly doses of SCIG may be higher than IV doses, SCIG products typically carry higher per gram prices than IV products, ancillary supplies for SCIG may be expensive, and facility/professional charges vary with location of infusions and in different provider networks. Interesting comparisons have been provided by Allen et al. (54) and Perraudin et al. (67) and report that the cost of the IgG product, which may differ in different health care systems, is the major determinant of the overall cost, regardless of the route of administration.

### 3.2 Adverse effects and precautions

Polyclonal IgG is generally regarded as a relatively safe medication with only rare serious adverse effects and few cumulative complications. Cochrane and other systematic reviews in CIDP and other neurologic diseases generally report that 25 to 50% of patients experience adverse effects with IVIG in ≤10% of overall infusions (43, 54, 68–70). These may be uncomfortable or unpleasant but are usually not physiologically significant, and do not often lead to discontinuation of treatment or withdrawal from studies. Headache, fatigue and/or mild general malaise are the most common complaints in nearly all studies, followed by chills and/or fever, myalgias and nausea. Most of these reactions are infusion-related and can be prevented or treated by slowing the infusion rate and avoiding higher rates in subsequent infusions (71, 72). Medications such as antipyretics, nonsteroidal anti-inflammatory drugs, and/or antihistamines may be helpful in preventing or treating these common AEs, and some physicians use pre-treatment with corticosteroids on infusion days. A Cochrane review of placebo-controlled studies of IVIG in CIDP concluded that there was little difference in the risk of serious adverse events- in the largest trial, with 113 subjects randomized to IVIG and 95 to placebo for 48 weeks, serious adverse events occurred in 5% of IVIg-treated subjects (0.8% of infusions) vs. 8% of placebo-treated subjects (1.9% of infusions) (43, 73).

Nevertheless, true anaphylaxis and anaphylactoid reactions may occur, particularly in IgA-deficient individuals (74). In patients known to have antibodies against IgA or with a history of hypersensitivity to IgA and/or blood products, IVIG should be avoided or given with extreme caution. Other types of hypersensitivity reactions may also occur.

All products licensed in the US carry an FDA-mandated “black box” warning for risk of thrombosis, renal dysfunction and acute renal failure. Recognition of these AEs over the years has led to changes in the manufacturing processes and formulation of the products to increase their safety. The risks of thrombotic adverse reactions have been largely attributed to increased blood viscosity at extremely high IgG or paraprotein levels and/or to the presence of vasoactive and prothrombotic clotting factors. Serious thrombotic events attributed to IgG therapy have included stroke and myocardial infarction (71, 72, 75). The risk of hyperviscosity can be reduced by assuring that patients are well-hydrated and/or fractionating the IVIG dose. Perhaps due to the avoidance of large bolus IV doses and/or neutralization of prothrombotic factors in the tissues before they reach the vascular compartment, SCIG has been associated with a greatly reduced incidence of thrombotic complications (75). Improvements in the manufacturing processes to reduce the content of activated FXIa and other prothrombotic proteins and screening products with thrombin-generating activity assays have also reduced the incidence of thrombotic complications, but their possible occurrence should still be considered in patients with histories of or risk factors for thromboses.

The renal complications were mainly observed with lyophilized IVIG preparations that were stabilized with sucrose, and were attributed to tubular damage/dysfunction caused by intracellular accumulation of that sugar (76, 77). Elimination of sucrose from the excipient, and the use of amino acids rather than sugars as stabilizers have reduced the incidence of renal complications (78), but caution should still be used when administering large protein loads to patients with any degree of pre-existing renal impairment, and all patients receiving IVIG should be well-hydrated before beginning the infusion.

Aseptic meningitis was reported in a 1994 study as occurring in 6/54 (11%) of patients receiving IVIG for neurological indications, including in 4/8 patients with histories of migraine (79). A more recent systematic review indicated that other studies reported lower incidences (68), and a recent review of reports to the FDA of AEs from all uses of IgG in children (<18 years) during the years 2001 to 2023 found that aseptic meningitis accounted for 2.7% of all clinically significant AEs (80). Sekul’s observation that 50% patients with histories of migraine developed aseptic meningitis may suggest increased sensitivity to vasoactive substances in the IVIG preparations of that era and/or may be due to the reaction of antibodies in the IVIG with antigens or autoantibodies within the CSF. It seems likely that the aseptic meningitis cases represent one end of a spectrum of a range from mild to severe headache, migraine, to frank meningismus and CSF pleocytosis. Aseptic meningitis is reported less frequently in more recent reviews (68, 70). When it does occur, this AE usually responds to over-the-counter analgesics and remits within 24–72 h with no sequelae (80). Assuring adequate hydration before and during high-dose IgG infusions, premedication with acetaminophen or ibuprofen and limiting the maximum infusion rate have all been employed as strategies to reduce headache and prevent aseptic meningitis.

Transfusion-related lung injury (TRALI) and hemolysis have been reported rarely as AEs of IgG treatment, particularly with high dose therapy. The actual incidence is difficult to estimate, because pharmacovigilance databases do not calculate a denominator such as the total number of patients or doses administered. Eight cases of TRALI were recorded in French pharmacovigilance databases between 2010 and 2016, and 9 additional cases were found in a systematic literature review (81). Wei (80) found 7 cases of TRALI in an FDA database of AEs from IgG in children, amounting to 0.2% of all clinically significant AEs. Thus, TRALI is estimated to account ≤0.5% of serious AEs due to IVIG, and is believed to be caused by anti-neutrophil or anti-HLA antibodies in donor plasma from which the IVIG is manufactured, although susceptibility factors in the recipients must clearly also be involved, since the incidence is so low.

Most adults with blood types other than AB+ produce isoagglutinins (antibodies against blood group substances they do not express). Thus, polyclonal IgG prepared from pooled plasma from donors with diverse blood types retain these antibodies, which may then bind to red cells in the recipients. Sensitization of red cells in recipients of both IV and SCIG may result in asymptomatic Coombs (direct antiglobulin) test positivity, occasional cases of clinically significant hemolytic anemia, and extremely rare episodes of acute severe intravascular hemolysis (82–85). Overall, the FDA estimates that some degree of hemolysis occurs in approximately 1.6% of IVIg recipients (85). Levine et al. (86) reported on a retrospective chart review of 166 patients on 12 months of stable maintenance therapy at a single infusion center for a variety of neuromuscular diseases (78 for MS, 60 for CIDP or MMN, 22 for MG, 10 for myositis). This cohort received 2,232 infusions at a mean dose of 1.2 gr/kg/mo (range 0.44 to 2.58). No patient suffered an acute hemolytic event or clinically significant hemolytic anemia, but 21% had a decline of ≥3 g/dL in hemoglobin concentration at some point during the year. Similarly, there were no episodes of acute renal failure but there was an average decline of 2% in GFR in the whole group, with 10.3% of patients experiencing a drop of ≥ 20%. No thrombotic events occurred.

Serious hemolytic complications are rare but may cause renal failure, severe anemia, and even death. Clinically significant or serious hemolysis is most commonly associated with non-O blood group recipients, particularly with blood groups A or AB receiving high doses of IVIG (1–2 g/kg/day). Pre-existing inflammatory conditions which activate reticuloendothelial system macrophages are known to increase the risk of acute severe hemolytic reactions to high-dose IVIG (84). Limits on anti-A and anti-B in IgG products are set by the FDA, but hemolytic events continue to occur due to widespread use of high-dose IGIV, certain manufacturing processes designed to increase the yield of IgG, and individual patient factors (71, 82, 85, 87). Both selection of plasma donors to avoid individuals with high isoagglutinin titers and use of immune-affinity columns to remove isoagglutinins from the pooled product have been shown to reduce the isoagglutinins in the final products (82), but these steps are not routinely employed by all manufacturers. If a patient requires transfusion because of hemolysis, or has had IgG-induced hemolysis in the past, careful cross-matching should be performed to avoid exacerbating the problem.

## 4 Mechanisms of action (MOA) of polyclonal IgG in autoimmune and inflammatory diseases

High-dose IgG is generally considered a broad spectrum immunomodulator which is beneficial in a wide variety of diseases (88, 89). *In vitro* and animal experiments give the impression that polyclonal IgG can affect almost every type of cell involved in immune responses and can modulate actions on diverse organs and tissues. Actual clinical experience, however, suggests that IgG is mainly effective only in diseases mediated by IgG autoantibodies and/or complement, and that it is ineffective in most diseases mediated by cellular immunity (29). For example, IVIG is minimally effective in most forms of MS (90, 91), but it is very effective in neuromyelitis optica (NMO) caused by anti-AQ4 antibodies (92, 93). IVIG rarely, if ever, alters the underlying immunoregulatory dysfunction in chronic conditions (94), and its effects often fade within a few weeks, as shown below. As such, it should not considered a “disease modifying” drug in Figure 2 and it must usually be dosed at monthly or even shorter intervals. Clinical observations, pharmacodynamics and other evidence suggest that high-dose polyclonal IgG most often acts by neutralizing or lowering concentrations of pathogenic autoantibodies, and/or by inhibiting effector functions such as C′ activation. Some small studies suggest that IVIG increases T-regulatory cells in GBS (95, 96) but the clinical significance of this is not clear.

Polyclonal human IgG can lower the concentrations of pathogenic autoantibodies by anti-idiotypic neutralization and by accelerating their catabolism. Inhibition of complement (C′), which amplifies the effects of antibodies, is the third major mechanism that contributes to the efficacy of high-dose IgG in autoimmune diseases. It is important to realize that these mechanisms may act together, and may have synergistic effects, depending on the amount and type of autoantibody in the patient and on whether C′ and/or Fc receptors contribute to the pathology. The ability of polyclonal IgG to affect autoimmune disease by multiple mechanisms simultaneously and without increasing susceptibility to infection makes it preferable to immunosuppressants in many cases (89, 97, 98).

### 4.1 Anti-idiotypic neutralization

Immunoglobulin antigen binding sites are formed by small “hyper-variable regions” on each pair of light and heavy chains. These arise by recombination of gene segments and spontaneous mutations, and are expressed on the surface of B-cells where they act as receptors. Upon exposure to an antigen, B-cells with complementary binding sites are selected and stimulated to secrete their particular antibodies. The antigen-binding site on that antibody may, in turn, seem “new” to the immune system, and may be seen as an antigen itself. Each unique antigen-binding site is called an “idiotype,” and an antibody which fits into or blocks the antigen-binding site of another antibody is called an “anti-idiotype” (anti-id) (89, 99, 100). In the 1980s, it was reported that IVIG could neutralize “inhibitors” of clotting Factor VIII (FVIII) in hemophilia patients receiving FVIII replacement and individuals with acquired hemophilia (13, 101). These “inhibitors” are in fact autoantibodies against FVIII and are neutralized by intact IVIG as well as its F(ab’)_2_ fragments, suggesting anti-id binding (13, 101, 102). Indeed, excess free anti-id antibodies appeared in the circulation when patients with autoimmune anti-FVIII recovered (13).

Three types of evidence favor a conclusion that anti-ids in IVIG are contributing to its efficacy in any given situation: 1. Inhibition of binding of autoantibodies to target cells or purified antigens in ELISA or other *in vitro* assays. Other effectors (complement or leukocytes) must be inactivated or absent to be sure that the anti-idiotype is responsible. 2. Immobilized anti-ids may adsorb autoantibodies from patient sera. In both of these types of studies, demonstration that F(ab’) or F(ab’)_2_ fragments have the same effect as intact IVIG provides confidence that the observed effect results from anti-id neutralization rather than Fc-Fc or other types of interactions. 3. The presence of dimers consisting of anti-ids bound to the corresponding autoantibodies in patient sera, and/or formation of dimers upon addition of IVIG to serum in *vitro* or upon IVIG treatment *in vivo*.

Direct neutralization of autoantibodies by anti-ids is illustrated by the report of Van Doorn et al. (20, 103) that binding of anti-neuronal antibodies from a CIDP patient to neuroblastoma cells *in vitro* was inhibited by IVIG and its F(ab’)_2_ fragments. The patient responded transiently to plasmapheresis as well as to IVIG with rapid improvement, but then weakness recurred, so he was eventually maintained on weekly IgG infusions. Every attempt at tapering-off the infusions was unsuccessful, demonstrating the failure of IVIG to affect the underlying autoimmune process. Subsequently, the repeated infusions had to be continued for years. Sera from 8/25 normal individuals and 9/12 individuals who had recovered from GBS also had this anti-id activity which blocked the patients’ acute-phase IgG from binding to neuronal cells (20). Lopez et al. further showed that normal human sera contained antibodies which blocked the binding of GBS patients’ antibodies to the ganglioside GM1 (104, 105). Usuki et al. (106) showed that anti-ids against anti-GD3 antibodies neutralized the latter and reduced weakness in an animal model of GBS. Malik et al. (107) and Di Egidio (108) also showed that binding of anti-GM1 from sera of MMN patients to the ganglioside was inhibited by both IVIG and sera from MMN patients who had recovered and lost their anti-GM1 antibodies, and Yuki and Miyagi (109), showed that IVIG and its F(ab’)_2_ fragments contained a protein epitope that blocked the interaction of cholera toxin with GM1 suggesting that an anti-id mimic of the antigen was responsible. On the other hand, Peng (110) suggested that blocking the binding of cholera toxin was mediated by the sugar side chains on the IgG molecules rather than an anti-id per se. Anti-id regulation of immune responses is now recognized as an important mechanism of peripheral tolerance. For example, Oak et al. showed that sera from a majority of 238 heathy normal individuals contained antibodies against the autoantigen GAD65 but that these were circulating bound to anti-ids which neutralized their activity. In contrast, individuals with type 1 diabetes or stiff person syndrome had much higher levels of free, unblocked anti-GAD antibodies (111). There are many other anti-ids in IVIG (112–114), usually confirmed by showing that F(ab’)_2_ fragments have the same neutralizing activity as intact IgG and/or by use of intact IVIG or F(ab’)_2_ fragments on solid phase immunoadsorbents to remove autoantibodies from plasma. Blank et al. (115) have even published protocols for enriching anti-idiotypes from IVIG preparations for use in particular diseases by the use of antigen-specific autoantibody affinity columns.

Neutralization of pathogenic IgG autoantibodies by anti-ids *in vivo* is illustrated by formation of IgG dimers (most likely id-anti-id complexes) (116). Circulating dimers increased following IVIG infusions in CIDP and GBS patients who improved after IVIG as compared to those who did not improve (114, 117). Acid-induced dissociation of the IgG dimers from post-IVIG treatment serum revealed that they contained anti-G1Qb in 2/2 Miller Fisher syndrome patients, antibodies that bound to peripheral nerve fibers in 4/10 CIDP patients and antibodies against acetyl choline receptors in an MG patient (117). Since polyclonal IgG contains the antibodies from thousands of healthy donors, it follows that it contains many different anti-ids (89, 112, 118). Pre-existing and newly-formed dimers in plasma pools used for IgG production are reduced by the low pH treatment and stabilizers now used by most manufacturers (8). Ritter et al. reported that sera from healthy control individuals contained a mean of 2.4 ± 1.3% of the IgG in the dimer fraction and that multiple lots of two different IgG preparations (with different stabilizers) contained 4.0 ± 2.3% and 4.9 ± 1.5% dimers (117).

### 4.2 Increased catabolism of IgG autoantibodies

To better understand this MOA, it is helpful to review the pharmacokinetics and pharmacodynamics of polyclonal IgG. Infusion of 2 g/kg of IVIG increases the serum IgG level *>*4-fold, from pretreatment means of 700–1,060 mg/dL to peaks well over 3,000 mg/dL (119). The levels then drop by about 50% over 48–72 h, as IgG is distributed into the total extracellular fluid volume, of which only 50% is intravascular (47, 48, 120). After this rapid equilibration, the IgG is catabolized with first-order kinetics, with the half-life dropping from ≥ 28 days in normal individuals to 10–12 days at the concentrations achieved by high dose therapy (Figure 3). In many CIDP and MMN patients, the clinical response mirrors the serum IgG levels. The course of the CIDP patient in Figure 4a shows a rapid increase in strength within 7–10 days after each IVIG infusion, but the response then plateaus and returns to the pre-infusion baseline within 2–3 weeks. This is diagrammed schematically in Figure 4B. Only after the true immunomodulators, prednisolone and azathioprine, were added towards the end of the third cycle did the baseline begin to show cumulative improvement over time. Figure 4A, towards right, after day 84.

**Figure 3:**
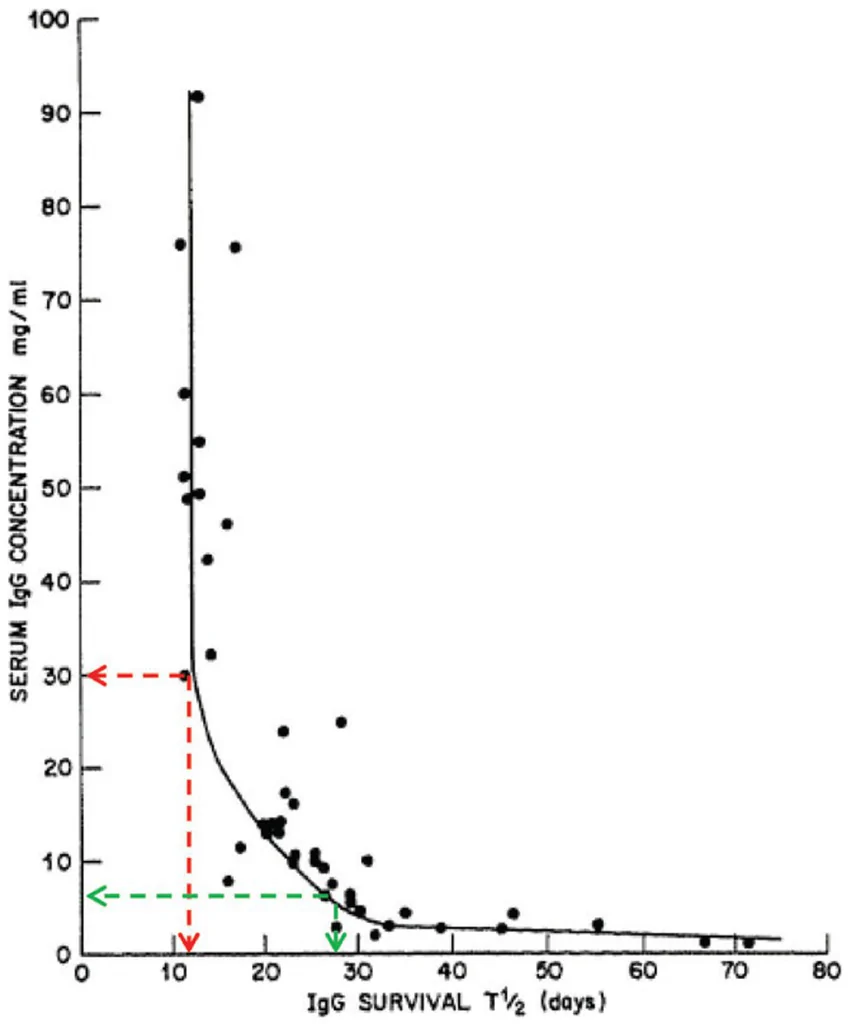
Effect of serum IgG concentration on IgG half-life. The half-life of IgG at normal serum concentration of about 800 mg/dl (8 mg/ml) is 28 days (green dashed line). Elevating the concentration to 3000mg/dl (30 mg/ml) reduces the half-life (increases the rate of catabolism) to 10-12 days (red dashed line). The concentration of 3000 mg/dl is achievable with high dose IgG. From: Waldmann TA, and Strober W (48). With permission from the publisher.

**Figure 4:**
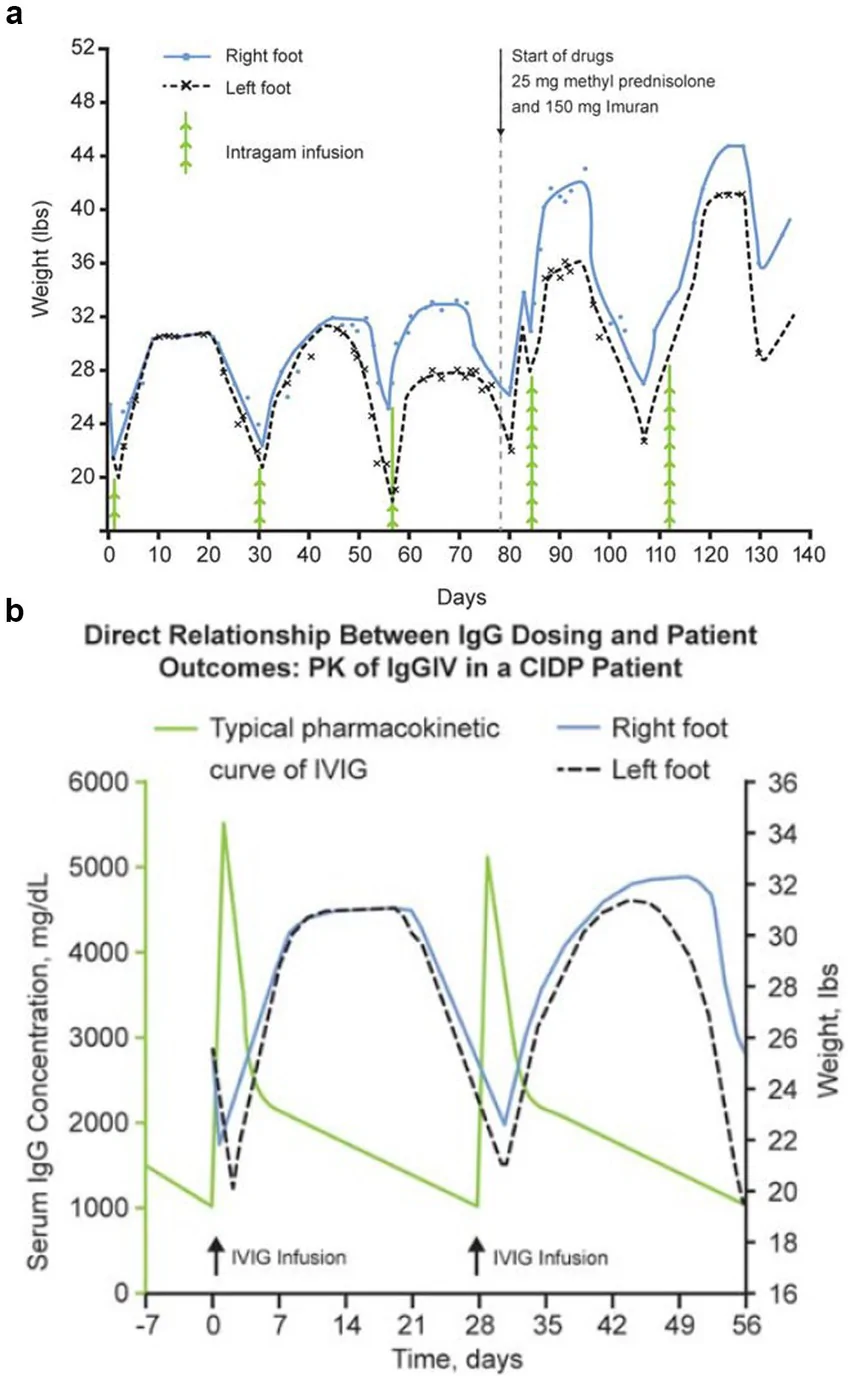
**(a)** Clinical response to treatment in a CIDP patient. y-axis shows strength of dorsiflexion in pounds lifted. Note cyclic gain in foot strength, and wear-off effect, in response to monthly intravenous immunoglobulin (IVIG) with little change in baseline to which she returns after each IVIG treatment. In contrast, baseline rises after addition of prednisone and azathioprine at day 79. (original patient data from Pollard and Armati, 2001). **(b)** Strength of foot dorsiflexion response to IVIG treatment of CIDP patient in Figure 4a (blue and dashed black lines, lbs shown in right axis)) superimposed on typical curve of pharmacokinetics of serum IgG after a dose of IVIG (green line, mg/dl on left axis) (original Pk data from Bonilla (47). Both from: Berger M and Allen JA (267). Open access use by Creative Commons agreement CC-BY.

**Figure 5:**
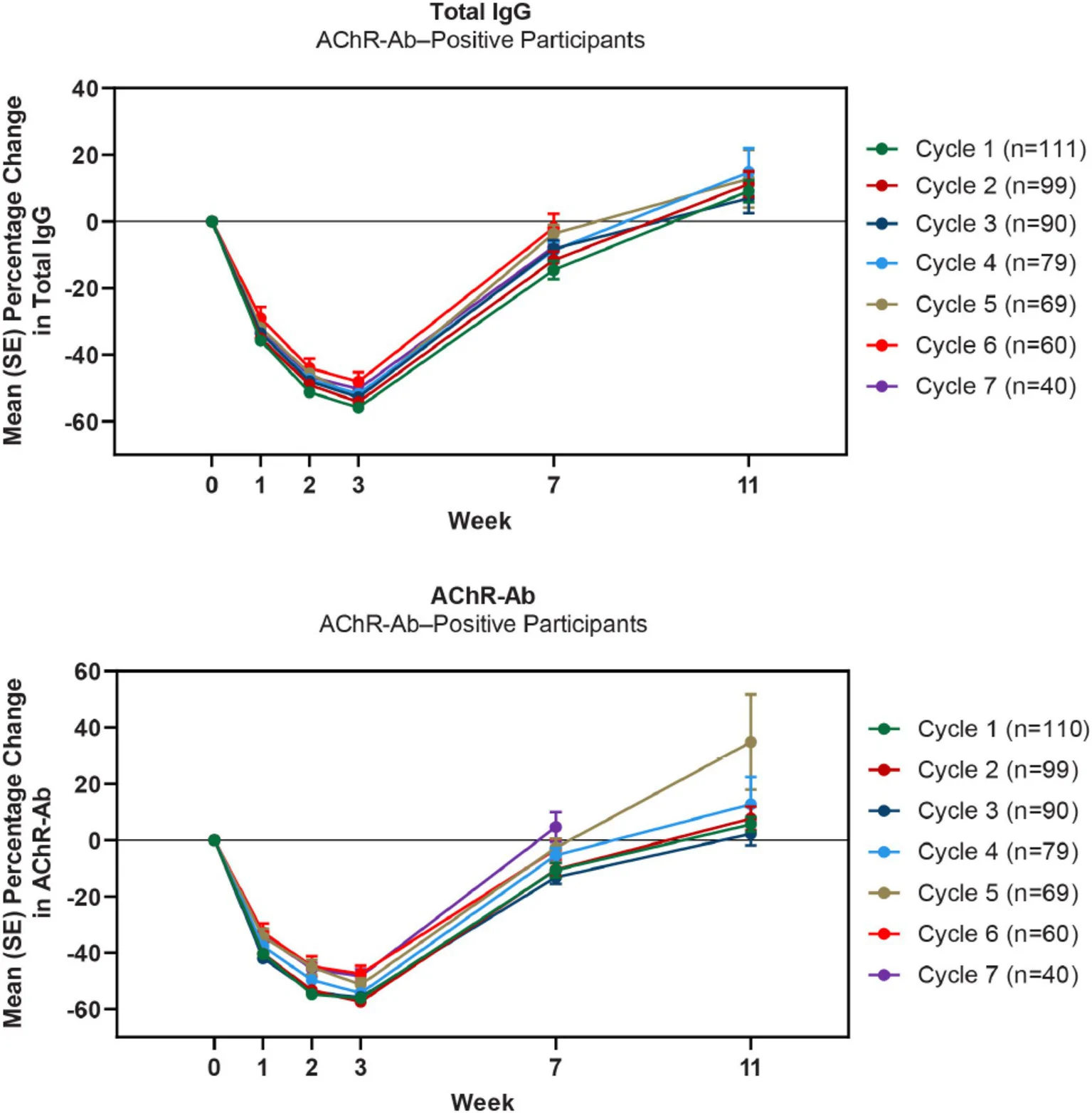
Effect of efgartigimod on total IgG (top) and anti-AChR (bottom) in anti-AChR+ Myasthenia Gravis patients. Different colored lines represent sequential treatment cycles. Each cycle consisted of four IV infusions of 10 mg/kg at weekly intervals, followed by an observation period of ≥ 4 weeks from last infusion. Next cycle was initiated when subject had MG-ADL ≥ 5 or no longer maintained clinically meaningful improvement from baseline (≥ 2 point reduction in MGL-ADL score). [from Howard et al. (140)]. Open access by CC-BY agreement.

The fluctuations in serum IgG levels and resulting fluctuations in therapeutic effect (decreased disability) can be evened-out by fractionating the IgG infusions into smaller increments given more frequently. This can be conveniently achieved by giving the IgG subcutaneously (SCIG) at weekly or even shorter intervals. The course of the MMN patient in Figure 2 provides an interesting illustration of this. After an apparent re-setting of his baseline by the first 2 months of IVIG therapy, he continued to have repeated cyclic “therapy-associated fluctuations” each month for the next 10 years. When his therapy was switched to weekly SC infusions, his strength became more stable, then increased and remained constant when the SC dose was increased (121). SCIG is associated with fewer systemic adverse effects than IVIG (55, 56) and can be safely self-administered at home, making it the preferred route for many patients (49, 54, 56, 122). A meta-analysis of 8 studies comprising 50 patients with MMN and 88 with CIDP found no significant differences in muscle strength outcomes (55). A randomized controlled trial comparing two doses of SCIG with placebo in 172 CIDP patients showed efficacy comparable to that previously shown with IVIG (123), and reviewed by Allen et al. (54). Based on those results, a 20% SCIG preparation was licensed by the FDA for CIDP in 2018, and 4 SCIG preparations are now licensed for CIDP (124).

Catabolism of IgG is controlled by a saturable Fc receptor on endothelial cells termed FcRn because it also transports IgG across the placenta providing antibodies for the newborn (125). IgG, together with other plasma proteins, is internalized by bulk fluid endocytosis, but FcRn specifically binds IgG and albumin at low pH and protects them from degradation in acidified lysosomes. The bound IgG is recycled back to the cell surface and released into the plasma at normal pH. This maintains the relatively high concentration and long half-life of circulating IgG (125–127). In contrast, IgA and IgM do not bind to FcRn (125) and have very short half-lives (128). In FcRn knockout mice and in the rare patients with FcRn mutations, the half-life of IgG is also very short, and its plasma concentration is quite low (129, 130). It is difficult to passively transfer IgG-mediated pathology to FcRn knockout mice because the half-life of the pathologic IgG is too short (131). When FcRn is saturated by high doses of exogenous normal IVIG, the catabolism of endogenous pathologic IgG is greatly increased (Figure 3) (48, 132). In wild-type mice, high dose IgG dramatically increases the catabolism of pathogenic IgG; while in FcRn knockout mice, high dose IgG does not further enhance the already rapid catabolism of pathologic IgG (131). Thus, by saturating FcRn with normal antibodies, high dose polyclonal IgG increases the degradation of pathogenic IgG autoantibodies. The underlying immunoregulatory abnormalities and autoantibody production are not affected however, and as the total serum IgG concentration falls back into the normal range, the autoantibody concentration rises and disease manifestations recur. In this sense, the effects of high dose IgG and plasma exchange are very similar- both remove pathogenic autoantibodies- but neither ameliorates the underlying immunoregulatory disorder or the rate of autoantibody production. Both must be repeated at regular intervals to maintain control of chronic conditions (133).

*FcRn blockers*: Recognition of the role of FcRn in autoimmune disease and the effects of high dose IgG in saturating it have led to the development of a new class of therapeutic agents, FcRn blockers. The efficacy of these blockers supports the conclusion that increased catabolism of pathogenic autoantibodies is a major mechanism of action of polyclonal IgG. FcRn blockers approved for neuromuscular diseases include efgartigimod, a human Fc fragment (MW ~ 51kD) engineered with 5 amino acid substitutions to increase its affinity for FcRn (134); rozanolixizumab, a single amino acid substituted intact humanized IgG_4_ monoclonal antibody which targets FcRn (135), and nipocalimab, a non-glycosylated human monoclonal which binds FcRn with high affinity at neutral as well as acid pH (136–138). All three of these are approved for generalized MG patients with antibodies to AChR or MuSK (Prescribing Information: ArgenX, Janssen, UCB). Efgartigimod with hyaluronidase (which facilitates subcutaneous administration and absorption) has also been approved for CIDP (FDA 6/24/2024) (257). Additional monoclonal anti-FcRn antibodies are also in late-stage clinical development. These agents do not discriminate between normal protective IgG and pathogenic autoantibodies-they reduce the concentrations of all IgGs. For example, in the efgartigimod “ADAPT” trial in MG, total IgG was reduced by a mean of 61% and anti-AChR titers were reduced by 58% (139) and Figure 5. In the major phase 2 study of efgartigimod, total IgG levels dropped by 70% by the 4th week and returned to baseline by the 10th week. Anti-AChR antibody levels decreased in parallel with the total IgG levels, declining by >50%, but did not return to baseline until after the 10^th^ week (140). The similarities in clinical response to the FcRn blockers and high dose IgG strongly suggest that they share a common MOA. As expected for agents that lower IgG antibody levels, infections have received considerable attention as potential AEs from the FcRn blockers. This has not proved to be a major problem so far, as indicated by individual clinical trials, a proportionality analysis of FAERS reports of 2,519 AEs with efgartigimod and 442 with rosanolixizumab (141), and a meta-analysis of reports of phase 2, 3 and open label extension clinical trial studies of efgartigimod vs. placebo including a total of 715 patients with MG, ITP or CIDP (142). Real-world clinical experience continues to accumulate (143–145), and periodic reviews of the data should allow continued surveillance of the infectious and other potential adverse effects of these agents. Headache and common types of respiratory and urinary tract infections have been amongst the most frequently reported adverse effects in clinical trials and reviews but have mostly been of mild or moderate severity and serious safety signals have not emerged [Prescribing Information: ArgenX, UCB, Janssen (139, 141, 142, 144, 145)]. Antibody levels and vaccine responses were assessed in patients with gMG who were treated with efgartigimod and who received influenza, pneumococcal, or coronavirus vaccines during participation in the efgartigimod trials. FcRn antagonism reduced levels of protective anti-TT, anti-VZV, and anti-PCP antibodies and total IgG to a similar extent, but the titers remained above minimally protective thresholds for > 80% of the patients and returned to baseline values after the treatment. Efgartigimod did not interfere with influenza, pneumococcal, and COVID-19 immunizations in patients who received them during the treatment period (146). Similarly, a meta-analysis of pre-existing antibody levels and vaccine responses in 3 studies of nipocalimib (one each in gMG, rheumatoid arthritis and Sjogren’s syndrome) reported that pre-existing titers of anti-tetanus toxoid and anti-herpes zoster IgG followed the kinetics of total IgG during nipocalimab treatment (60–65% reduction in antibody titers and 53–68% reduction in total IgG levels, respectively) and returned to baseline after discontinuation. However, the majority of nipocalimab-treated participants maintained levels of these antibodies above the threshold level for protection. As compared with placebo, nipocalimab did not interfere with IgG responses to SARS-CoV-2 vaccination, and SARS-CoV-2 infections during the studies were mild to moderate with no complications (147).

All three approved FcRn blocking agents carry “Warnings and Precautions” about infections, hypersensitivity reactions and infusion reactions, but none carry “Black Box Warnings” (Prescribing information) (255, 256, 261, 265).

### 4.3 Inhibition of complement (C′) by high dose IgG

C′ is a cascade system in which inactive precursors become activated by proteolytic cleavages and conformational changes to combine into multi-subunit enzymes called “convertases” which activate many molecules of the next protein in the sequence, achieving amplification (148, 149). Binding of multiple Fc regions of IgG or IgM to the hexavalent C1q molecule initiates the “classical pathway” of activation leading to conversion of the latent proteases C1r and C1s to their active forms. C1s cleaves C4 and C2, which then form “C3 convertase” that cleaves C3 into C3a and C3b. C3b is a key subunit of the alternative pathway C3 convertase and all of the C5 convertases. This activity of C3b is most efficient when it is attached covalently to the target, providing an anchor for the other subunits. Promotion of C3 convertase formation by C3b activates the alternative pathway to serve as an amplification loop, and many molecules may become attached to the target. Macrophages, neutrophils and other inflammatory cells have receptors for C3b and its derivative iC3b which promote attachment of those cells to the target. This may be important in promoting attack of macrophages on nerves in the demyelinating neuropathies. Activation of C5 leads to formation of the C5b-9 membrane attack complex (MAC), a protein-lined pore which can insert into and damage target cell membranes. Although insertion of a single MAC pore can lyse red cells, nucleated cells may pinch-off and remove patches of membrane bearing MACs. In doing so, however, the target cell may lose functionally important membrane structures such as AChR from motor endplates in MG, or adhesion molecules and ion channels near nodes of Ranvier.

High dose IgG provides an excess of favorable attachment points for C3b, competing with the target and thus decreasing continued C′ activation and tissue damage (150–152). Inhibition of C3b deposition also inhibits C′ receptor-mediated attack by macrophages and neutrophils. This “complement scavenging” activity is likely the major mechanism of action by which high-dose polyclonal IgG inhibits further C′ activation and ameliorates cellular damage/dysfunction in MMN, and it is also an important MOA in dermatomyositis, MG and GBS (153). IVIG has also been reported to bind the chemoattractant and pro-inflammatory peptide C5a (154), but the significance of this *in vivo* is not clear. The pathogenesis of MMN likely involves IgM rather than IgG, and several studies report finding antibodies against GM1 and/or GM2 in sera from MMN patients [(155, 156), and see below]. IgM is a particularly good activator of the classical pathway of C′ because its multiple Fcs can bind simultaneously to a single C1q molecule. Yuki et al. (156) showed that IVIG inhibited MMN patient sera-induced C4b deposition and C′ activation on GM1-coated ELISA plates. Recent studies with *in vitro* cellular models confirm that MMN patients’ sera contain anti-GM1 and cause C′ activation by the classical pathway which can be inhibited by IVIG and antibodies against C1q, C2 and a synthetic inhibitor of C3, but not by two different anti-C5 antibodies (157). Anti-C1s and Anti-C2 also inhibited C′ activation by MMN patients’ sera in these models ((158, 159), respectively).

*Complement* *inhibitors*: The efficacy of C′ inhibitors in neurologic diseases suggests the importance of inhibition of C′ deposition/activation as an MOA of polyclonal IgG (153). Eculizumab and raviluzumab are IV-administered monoclonal antibodies against C5 which prevent its cleavage and therefore inhibit formation of the MAC and release of the chemoattract C5a (160, 161), respectively. Inhibition of MAC formation may decrease membrane and receptor loss (226, 227). Zilucopan is a 15 amino acid macrocyclic peptide which has similar effects. Zilucopan can be self-administered by a once-daily subcutaneous injection from a pre-filled syringe (UCB Zilbrysq Prescribing Info) (266). All three of these C′ inhibitors are approved for generalized MG, attesting to the importance of C′ and the MAC in the pathogenesis of MG (162, 163). Like individuals with genetic deficiencies of C5 and other MAC components, patients taking these inhibitors are particularly susceptible to serious and life-threatening infections with *Neisseria* spp., including meningococcal sepsis and meningitis (FDA “Black Box” warnings in prescribing information), and should be immunized with meningococcal vaccine before receiving C′ inhibitors (164). A recent systematic review of 9 randomized controlled studies of eculizumab in diverse diseases included 2 studies each of its use in MG and GBS, totaling 230 subjects with these diseases. The overall rate of infection was not significantly different between eculizumab and placebo groups (47.2% vs. 45.7%, respectively. Total *n* = 548 subjects), although there were increased incidences of urinary tract infections and bacteremia in the eculizumab treated subjects (165).

Other C′ inhibitors which have shown promising results in early phase clinical trials and are now in phase 2 or phase 3 registration trials include tanruprubart, a monoclonal antibody against C1Q in GBS (NCT 07020819, phase 3); claseprubart and riliprubart, monoclonal antibodies against C1s, both in phase 3 for CIDP (NCT 0685879 and NCT 06290128, respectively); and empasiprubart in MMN (NCT 05225675 and NCT 05405361, both phase 3).

Zilucopan has completed or is currently in multiple different phase 3 and/or open label extension trials in different age- and clinical-sub groups of MG patients (NCT06471369, NCT07215949, NCT06511076, NCT06435312, NCT05514873, NCT04225871, NCT04115293). In the major placebo controlled phase 3 trial (NCT04225871), 86 subjects received zilucoplan, 0·3 mg/kg, and 88 placebo. Zilucoplan showed a significantly greater reduction in MG-ADL score from baseline to week 12 as compared to placebo. Treatment emergent adverse effects occurred in 66 (77%) patients in the zilucoplan group and in 62 (70%) patients in the placebo group, the most common being injection-site bruising (*n* = 14 [16%] in the zilucoplan group and n = 8 [9%] in the placebo group). There were no differences between the groups in serious infections or other serious AEs (166). In the open-label extension, NCT04225871, zilucopan demonstrated a favorable long-term safety profile, good tolerability, and sustained efficacy through to week 60 with consistent benefits in a broad AChR+ gMG population (167). In a real-world study in France of 48 MG patients receiving Zilucopan in an early-access program, 3 patients discontinued zilucoplan due to lack of efficacy and 4 discontinued the drug because of adverse effects. However, in treated patients, mean MG-ADL and QoL scores improved and 9/43 patients achieved minimal symptom expression at month 3 and 12/30 at month 6. The mean oral corticosteroid dose decreased from 28.8 ± 11.8 mg to 17.9 ± 11.8 (*p =* 0.001). Only 6 patients experienced MG crises on treatment, compared to 22 in the 6 months preceding enrollment (168).

In contrast to these inhibitors of C5 and the membrane attack complex, the alternative pathway inhibitor (anti-factor D monoclonal antibody) vemircopan failed to show better efficacy than placebo in a phase 2 RCT in MG (169), and eculizumab failed to meet its primary and secondary endpoints in a phase 3 RCT in GBS (170). A small clinical trial of anti-C5 in MMN showed no effect in attenuating symptoms (171), but that may be consistent with the actions of C′ (and IVIG inhibition) in depositing C3 fragments, and activating macrophages being more important than activity of the MAC in this particular physiologic situation.

## 5 Brief review of major neurologic/neuromuscular diseases treated with polyclonal IgG

### 5.1 CIDP

CIDP is characterized by symmetric proximal and distal weakness, with sensory loss and decreased deep tendon reflexes. There are also several atypical or “variant” presentations (41). The global prevalence of CIDP is estimated at 8–9 per 100,000 individuals ((172, 173), GBS/CIDP Foundation) (258). The course in individual patients may vary with time, but permanent disability can occur. Evidence for a primary role of humoral immunity in CIDP pathogenesis includes detection of IgG, IgM and/or complement in nerve biopsies (174), *in vitro* antibody staining of nerve fibers (174) and passive transfer using sera or purified IgG from patients (175). However, anti-neuronal antibodies are not usually detected by current assays, and demonstration of autoantibodies is not required by current consensus diagnostic criteria (41). Histopathology studies of C′ deposition in sural nerve biopsies from 55 CIDP patients revealed positive staining for C4d in 9/9 CIDP patients, and C5b-9 in 52 (94%) [(176, 177), respectively], consistent with an important role for antibodies and the classical pathway of C′.

There are no widely-accepted objective biomarkers of CIDP disease activity, so clinical function/disability assessment tools are used as endpoints in clinical trials (178–182). The most recent Cochrane review of IVIG therapy in CIDP includes nine RCTs with 372 participants: IVIg compared with placebo increases the probability of significant improvement in disability and there was little or no difference between IVIG and placebo in the frequency of serious adverse events (43). Several systematic reviews found only very limited data (from small cross-over studies) directly comparing IVIG with plasma exchange, with no apparent differences in efficacy (43, 69, 183). Plasma exchange (PLEX) and high dose IgG have shown efficacy and similar kinetics of onset and duration in separate studies, and both, as well as corticosteroids, are considered “first-line” treatments (41). However, high dose IgG predominates because of its relative ease of administration and lack of long-term adverse effects (3, 41, 44), but SCIG can also be used.

FcRn blockers have also been approved for CIDP (161, 184, 185, 257), but clinical experience with these is limited. Several C′ inhibitors are in late-stage clinical development (263). A recent survey of “real world evidence” which included data on 1,056 CIDP patients reported that 55.7% were prescribed immunoglobulins (IV/SCIg) and 44.5% corticosteroids. For the total cohort, there were only 14 reported uses of plasma exchange therapy. Non- steroidal immunosuppressants, monoclonal antibodies, and other treatments, such as neuropathic pain therapies, were prescribed to 33.6% of patients (186). Two studies of rituximab in CIDP have given conflicting results (187, 188), most likely due to differences in the patient populations, whose pathologic antibodies were not characterized. A 2022 systematic review of 96 subjects across 15 studies found that 75% responded to rituximab (189). Larger studies, in subjects whose antibodies are known to be of the IgG_4_ vs. other subclasses, or who have clearly failed to respond to an optimized regimen of high dose IgG, will be necessary to define the potential role of rituximab in CIDP and identify those patients most likely to benefit from therapies which target antibody production *per se*.

*Guidelines and Summary:* For CIDP, an EAN/PNS task force (41) “strongly” recommended initial therapy with IVIG or corticosteroids, but considered the evidence insufficient to express a preference for the route of steroid treatment (oral vs. IV) or choice of drug (prednisolone/prednisolone vs. dexamethasone). IVIG was recommended as a first line treatment for motor CIDP and for patients who deteriorated after initial trials of steroids. IVIG and SCIG were recommended for maintenance therapy, but SCIG was not recommended for initial induction. PLEX received only a “weak” recommendation. Use of methotrexate, interferon-beta and fingolimod were not recommended, and other immunosuppressives such as cyclophosphamide, cyclosporin and rituximab were recommended only after failure of first-line treatments.

IVIG, SCIG, and FcRn blockers have been all approved for CIDP, but comparisons of the latter with IgG are not available and have not yet found their way into guidelines or consensus statements. Late-stage clinical trials of C′ inhibitors have not yet been completed.

### 5.2 Nodopathies

An important exception to efficacy of polyclonal IgG in GBS and CIDP is provided by the small subset (≤10%) of patients previously included under the umbrella of CIDP who have now been diagnosed with what is now termed “autoimmune nodopathy” (41, 190–193). These patients stand out clinically as being older and with an aggressive course that prominently features tremor, ataxia and pain, and “poor responsiveness” to IVIG. Laboratory analyses and animal studies suggest that these patients have neither demyelination or inflammation, but unlike most CIDP patients, they do have detectable anti-neuronal antibodies. The antibodies target adhesion and structural proteins found in and around the nodes of Ranvier, including contactin 1, contactin associated protein (CASPR1), neurofascin 155, and pan-neurofascin (186, 190, 191, 262). These proteins are necessary for proper attachment of myelin to the axon and/or localization and action of clustered ion channels. The antibodies are predominately of the IgG_4_ subclass, which do not activate complement and have lower affinities than other subclasses for Fc receptors on myeloid effector cells (194–196). It is not clear why these patients respond poorly to IVIG, but at least part of the resistance is due to the fact that IgG_4_ activates C′ poorly, thus limiting the role of C′ activation as part of their pathophysiology (190–193). Correspondingly, blocking C′ would offer limited relief. It has been proposed that IgG_4_ antibodies have low affinity for FcRn, and hence their serum concentrations are not reduced by polyclonal IgG competition for FcRn or by FcRn blockers, but this has not been tested directly. West and Bjorkman (197) reported that homogeneous myeloma proteins of all 4 subclasses had equilibrium dissociation constants for FcRn in the nanomolar range but within the same order of magnitude. The decreased affinity for FcRn may also contribute, and the ability of IgG4 to exchange arms and therefore to have two different monovalent antigen binding sites (198) may weaken anti-idiotypic binding and may also affect binding to FcRn. Interestingly, Anti-MuSK antibodies in myasthenia gravis are also reported to be predominately in the IgG_4_ subclass. Patients with these antibodies do respond to IVIG, although it seems somewhat less effective than PLEX. On the other hand, FcRn blockade has proven quite effective and several of the FcRn blockers have been approved for use in MuSK-positive in addition to AChR+ MG patients. Fortunately, the IgG_4_ antibodies important in nodopathies seem to be mainly produced by plasmablasts, rather than mature plasma cells. This is important because B-cells and plasmablasts retain CD20, which is lost upon full maturation to plasma cells. Their expression of CD20 makes them susceptible to anti-B cell therapies such as rituximab, which is very effective (199) and is the treatment of choice for these IgG_4_ mediated neuropathies (188, 192).

### 5.3 Multifocal motor neuropathy (MMN)

MMN is characterized by progressive, asymmetric weakness and muscle atrophy, generally affecting the upper extremities more than the lower. The most common symptoms are weakness, cramping, involuntary contractions or twitching, and muscle wasting (200–202). MMN is much less common than CIDP, with a global prevalence of only 0.6 to 2 cases per 100,000 (GBS/CIDP Fdn.). Electrophysiologic studies may show conduction block in addition to slowing. IgM antibodies against the ganglioside GM1, which is more highly expressed in motor than sensory nerves, have been detected in one third to half of MMN patients (155, 203–205). IgM is a very efficient activator of the classical pathway of C′, and C′ activation by MMN patients’ sera *in vitro* has been correlated with anti-GM1 antibody titers and disease activity (206). Activation and deposition of C3b may promote macrophage attack on nerves, and the C′ membrane attack complex (MAC) may also damage the axonal membrane leading to loss of critical structures, somewhat analogous to the loss of acetyl choline receptors (AChR) in MG. Interestingly, and unlike most other immune mediated neuropathies, plasma exchange (PLEX) is not effective, and may even worsen MMN (45, 123, 207–209). Indeed, polyclonal IgG is the only approved and recommended therapy (26, 40, 155, 201, 202, 210). The lack of efficacy of PLEX suggests that decreasing autoantibody titers is not a major mechanism of action of high-dose IgG in MMN, since the responsible antibodies are likely IgMs. Recent attention has focused on C′ inhibition.

### 5.4 Myasthenia gravis (MG)

MG is a prototypical antibody-mediated autoimmune disease (reviewed by (211)). IgG autoantibodies against acetylcholine receptors (AChR) on muscle motor end plates are present in 80–85% of MG patients (211–213). Anti-AChR antibodies have multiple effects at the neuromuscular junction: blockade of neurotransmitter binding; cross-linking, clustering and clearance of the receptors; and activation of complement (C′) (214, 215, 259). Formation of the C′ MAC on the post-synaptic membrane may lead to removal of damaged membrane with corresponding loss of receptors and related proteins (140, 216). Transfer of anti-AChR antibodies across the placenta may cause transient weakness in the newborn (217). Among patients who lack anti-AChR and were previously regarded as “seronegative,” other autoantibodies which target post-synaptic membrane proteins such as muscle-specific kinase (MuSK), low-density lipoprotein receptor-related protein 4, and agrin have been identified and have similar effects. The underlying autoimmune process is related to thymoma in some cases, thus thymectomy may be effective in treating MG (218).

Investigations shortly after the introduction of IVIG showed that it was effective in MG. Like plasma exchange (PLEX) (219), IVIG also decreased AChR antibody titers in many patients (14, 16). Many studies, meta-analyses, and Cochrane reviews found no significant differences in efficacy between IVIG and PLEX for generalized MG (183, 220, 221). These studies generally include stable AChR+ patients on maintenance therapy. A 2021 meta-analysis restricted to acute MG found 4 qualified studies comparing PLEX with IVIG in 292 patients. The pooled estimate based on a random effects model was a + 19% difference in response in favor of plasma exchange vs. IVIG (*p* = 0.002). Further, a recent systematic review of 14 studies by Ghimire et al. (220), PLEX gave a higher odds ratio of any improvement as compared to IVIG, but severity scores did not differ significantly between the two treatment groups. Interestingly, elevated titers of anti-MuSK antibodies, likely of the IgG4 subclass, showed greater efficacy of plasmapheresis compared to IVIG (220). In a retrospective analysis of 110 AChR- MuSK + patients, PLEX had been used in 72 patients with physicians reporting improvement in 93%, while IVIG was used in 28% with improvement reported in only 61% (222). Multiple guidelines recommend that choices between the two therapies be based on individual patient or institutional considerations (40, 220, 223–225). FcRn blockers mimic the action of polyclonal normal IgG in decreasing antibody titers in AChR+ MG and improving weakness (139, 140, 226). Efgartigimod, rosanlixizumab and nipocalimab are now all licensed for both AChR+ and MuSK+ generalized MG (ArgenX, UCB and Johnson and Johnson package inserts).

C′ inhibitors that block formation of the MAC are also effective (160–162, 227), suggesting that C′ inhibition also contributes to the efficacy of polyclonal IgG in MG. Studies with the FcRn blockers and these agents (228–230), emphasize the importance *in vivo* of increasing catabolism of autoantibodies and inhibiting C′ as compared to other putative MOAs of high dose polyclonal IgG.

*Rituximab*: Rituximab is a monoclonal antibody against CD20, which is expressed on B-cells and some plasmablasts, but not on fully committed plasma cells. It causes depletion of B-cells by activating complement on the B-cell surface and by antibody dependent cellular cytotoxicity. It was first used 30 years ago for B-cell malignancies and has been widely touted as a “promising” new agent for antibody mediated autoimmune diseases, but it is not licensed in the US for any neuromuscular disease. Because rituximab does not affect antibodies that have already been secreted by B-cells, its clinical effects may become apparent only after weeks or months (264).

A systematic review in 2024 reported a meta-analysis of 17 studies involving 292 MG patients. Both AChR+ and MuSK+ patients were included in the studies. A noticeable improvement in clinical status was observed in 91% of patients at the final follow-up after a mean of 23 months of therapy (95% CI: 84–96%, *p* < 0.001) (231). In contrast, however, a recent Cochrane review found only two studies suitable for pooled analysis out of 6,891 records identified in electronic literature searches concluded that rituximab’s effects on MG symptom severity and functional ability remain uncertain. Rituximab resulted in little to no long-term steroid-sparing effect at 9 months, but two studies suggested it resulted in a large reduction in relapse requiring rescue therapy over 9 months, but that conclusion was based on only two suitable studies. The overall conclusions were that data was inadequate to determine optimal dosing regimen or patient characteristics (232).

*Guidelines and summary for MG*: Thymectomy is often indicated in MG and has been shown to be beneficial in a large, multicenter controlled study (218). For medical therapy, international consensus guidelines recommend pyridostigmine and corticosteroids as first-line treatments, with PLEX, immunomodulators and immunosuppressives reserved for patients with acute exacerbations (impending crisis) insufficient responses or intolerance (225). UpToDate notes that most patients with generalized MG will also require immunotherapy at some point (233). PLEX and IVIG are considered appropriate for short-term treatment in patients with MG with life-threatening signs such as respiratory insufficiency or dysphagia; in preparation for surgery, when a rapid response to treatment is needed or when other treatments are insufficiently effective. The guidelines noted that IVIG and PLEX are probably equally effective in for severe generalized AChR MG. However, some reports suggest that PLEX might be more effective in patients with anti-MuSK antibodies, and rituximab has also shown promise, but has not yet been considered for approval. IVIG is generally considered for maintenance therapy in those patients with refractory MG or in whom other steroids and immunosuppresive agents are relatively contraindicated. The complement inhibitors eculizumab and ravilizumab are approved by the FDA for generalized MG (160, 161) but are not usually considered first-line agents (223, 224, 226, 227). FcRn blockers have been approved for MG, and seem relatively safe, but their place in long-term management has not yet been determined.

### 5.5 Guillain-Barre Syndrome (GBS)

GBS is actually a family of acute, monophasic, immune-mediated disorders primarily affecting peripheral nerves. Reported incidences vary from 0.16 to 3 cases per 100,00 persons/year, depending on age and geographic region [(234–236), GBS/CIDP Foundation] (258). Typical features include rapidly progressive, ascending, symmetric muscle weakness and absent or depressed deep tendon reflexes (42, 237, 238). Many patients complain of pain in the back and/or legs, often with tingling sensations or other paresthesias. Autonomic nerve involvement may cause fluctuations in blood pressure and heart rate, and cranial nerve involvement can result in facial weakness, difficulty with speech or swallowing, and aspiration or respiratory difficulty. Ascending paralysis may progress to hypoventilation and frank respiratory failure in up to 20% of patients if not treated promptly (238). Symptoms usually progress over 1–2 weeks and plateau by 4 weeks, followed by a slow recovery phase (238, 239). Progression or persistence beyond 4 weeks suggests other diagnoses (238, 240, 241). CSF typically shows “albuminocytologic dissociation” -elevated protein concentration without increased white blood cells. More than two-thirds of patients report a history of upper respiratory tract or gastrointestinal infection 1–2 weeks before the onset of neurologic symptoms, and GBS is frequently cited as an example of “molecular mimicry,” especially after infection with *Campylobacter jejuni* (238, 242). GBS has also been reported after some influenza vaccines (242, 243). Different forms of GBS are believed to result from targeting different gangliosides or neuronal protein antigens (238, 244). Mechanisms of antibody-mediated damage include demyelination, attack on axonal proteins and/or interference with ion channel function. Complement deposition has been demonstrated and is believed to compound antibody-mediated nerve injury. Macrophages as well as cytotoxic T-cells may also be involved.

*Treatment of GBS*: IVIG and PLEX are the only proven treatments for the acute stage of GBS. Both are instituted as quickly as possible to minimize nerve injury and prevent respiratory failure. Both act rapidly and are considered equally effective (183, 245, 246, 260) suggesting that both act primary by removing the pathogenic autoantibodies (238, 239). In general, the decision between IVIG and PLEX is dependent on local availability and costs. Studies by the Dutch group et al. have shown that the efficacy of IVIG correlates with the serum IgG level achieved, and that adding a second course of IVIG after an initial series of infusions totaling 2 g/kg has little additional effect (247). For GBS, the EAN/PNS task force recommendation (42, 241) is to begin IVIG or PLEX as soon as possible, with no preference between the modalities. Use of corticosteroids, other immunosuppressives or complement inhibitors was not recommended.

*FcRn Blockers in GBS*: Small initial studies suggest that FcRn blockers may be effective in GBS. Zhang et al. compared efgartigimod with IVIG and reported no difference in the proportion of subjects reaching a GBS-DS functional grade ≤2 at 4 weeks post-treatment: 3 of 9 (33.3%) in the efgartigimod cohort and 3 of 12 (25.0%) in the IVIG cohort. Similarly, there were no differences in the proportion of patients who improved ≥1 grade in GBS-DS at weeks 4 or 24 or in the GBS-DS grade at week 4. However, the MRC sum score demonstrated significantly greater muscle strength improvements in the efgartigimod cohort than in the IVIG cohort at weeks 1, 4 and 24. Serum IgG levels decreased by 65.53% (SD: 2.67%, *n* = 8) reduction in the efgartigimod cohort at 1 week post-treatment, but grade 3 lung infections occurred in 3 subjects in each group (248). A small observational study comparing efgartigimod with IVIG (11 patients in each group) found no difference in the proportion showing improvement in GBS-DS at weeks 1 or 2 (249). A retrospective observational study by Cheng included 5 patients treated with efgartigimod, 8 with IVIG and 4 with PLEX. Improvement by 1 point in the GBS-DS was faster in the efgartigimod group (4 days) compared to 7 days for IVIG and 11.5 days for PLEX, and improvement in INCAT and MRC scores at 1 week was also greater with efgartigimod (250). Results of a retrospective observational study of 20 patients treated with IVIG and 16 with efgartigimod by Tao et al. were also similar. Treatment emergent AEs were reported by 3 efgratigimod-treated patients and 10 IVIG-treated patients (251). Additionally, Kang et al. (252) recently published a review of 9 publications which together reported favorable results from treatment of GBS in 16 patients with efgartigimod, including some who were considered “refractory” to IVIG or PLEX. Taken together, these results suggest that rapid reduction of IgG autoantibodies makes a major contribution to the efficacy of IVIG in GBS, and suggests that FcRn blockers should be studied in larger, prospective randomized trials.

## 6 Summary and conclusions

Plasma-derived polyclonal IgG is effective in a wide range of autoimmune and inflammatory diseases. It is most effective against antibody and/or C′ mediated autoimmune diseases and it does not have significant efficacy in diseases in which cell-mediated immunity predominates (29). Increasing catabolism of IgG autoantibodies, anti-idiotypic neutralization and C′ inhibition are its major modes of action *in vivo*. IgG does not have major disease-modifying activity against the underlying autoimmune processes, however, so large doses must be given repeatedly. It is the mainstay of therapy in several chronic neurologic and neuromuscular diseases, especially CIDP and MMN, and its usage for those diseases is now equal to or greater than for immune deficiencies. IVIG is usually given at 3–4 week intervals, but individualization of doses and intervals may be helpful. Frequent subcutaneous infusions of highly concentrated (20%) IgG have been shown to be as effective as IV-administered IgG (55, 56). Frequent SC administration can maintain therapeutic IgG levels continuously, and minimize treatment-related fluctuations in strength and mobility. Because IgG does not alter the underlying autoimmune diathesis, therapy is often required for years.

High dose polyclonal IgG is uniquely effective in Kawasaki syndrome and toxic-shock syndromes, presumably because it contains antibodies which rapidly neutralize the toxic super-antigens that are believed to be responsible for these conditions (31, 253). Similarly, polyclonal IgG contains many anti-idiotypic antibodies which have been shown to neutralize a broad range of autoantibodies. In many of the autoimmune neuromuscular diseases, the multiple simultaneous actions of IgG favor its use for empirical therapy because autoantibodies, complement and other mediators are not usually identified in individual cases and it has a rapid onset of action. This is particularly important in GBS and CIDP, in which delaying therapy may increase nerve damage and disability. With the important exception of MMN, most neuropathies in which IgG is effective also respond to PLEX, but in many situations IgG is preferred because it is more convenient and does not require specialized equipment. Compared to other modalities of therapy, polyclonal IgG is unique because it is not broadly immunosuppressive and does not increase the risk of infection. Although it is expensive due to the requirement for paid donor plasma and high manufacturing costs, risks of true allergic sensitization are low, as are the risks of loss of efficacy by induction of anti-drug antibodies.

The anti-B cell monoclonal antibody rituximab has shown efficacy in CIDP and other neuro-muscular diseases, and it is particularly effective in the IgG_4_ mediated “nodopathies” (199), but its place in our armamentarium remains incompletely defined (264). Additional monoclonal antibodies with activity against fully differentiated plasma cells are being introduced for myeloma and other malignancies and have yet to studied in peripheral neuropathies, although they are finding niches in the treatment of multiple sclerosis. Newer therapies such as FcRn and C′ inhibitors replicate individual MOAs of polyclonal IgG and have proven effective in many patients. The anti-C5 monoclonal antibodies ravalizumab and eculizumab and the C5 analog zilucopan have been approved for generalized MG but their position in clinical care algorithms has not been firmly established and late-stage clinical studies of inhibitors of more proximal complement components have not yet been completed. Outcome measures in the neuropathies are difficult to quantitate, and there is considerable patient-to-patient heterogeneity, so it is not possible to estimate how much any single MOA of IgG contributes to its overall effect in any given patient. Randomized, controlled, head-to-head comparisons of different modalities are difficult and rare, but comparative reviews do help to delineate which of the multiple actions of IgG are most important in different disease states. Since IgG acts by multiple mechanisms simultaneously, not all patients who respond to IgG respond to these newer, more-selective agents, and there have been reports that some CIDP patients deteriorated rapidly when transitioned from IgG to FcRn blockers (254). On the other hand, most patients with IgG4-mediated nodopathies and some with anti-MuSK MG respond poorly to IgG but have shown favorable responses to rituximab and PLEX. While the use of polyclonal IgG continues to increase, studies with newer agents that target individual pathways should help us to better understand the pathophysiology of autoimmune neurologic and neuromuscular diseases, and may allow more precisely targeted precision therapy for some individual patients.

## Footnotes

1
https://www.fda.gov/vaccines-blood-biologics/approved-blood-products/immune-globulins

## Glossary

AChR: Acetyl choline receptor
Anti-id: Antibody against idiotypic determinant on another antibody
C′: Complement
CIDP: Chronic Immune Demyelinating Polyneuropathy
EAN/PNS: European Academy of Neurology/Peripheral Nerve Society
EMA: European Medicines Agency
FAERS: FDA Adverse Event Reporting System
Fc: Crystallizable fragment of antibody
FcRn: Neonatal Receptor for Fc of IgG (also found on endothelial cells in adult humans)
fSCIG: Subcutaneous immunoglobulin facilitated by hyaluronidase pre-treatment
GAD65: Glutamic Acid Decarboxylase (65 KD)
GBS: Guillain-Barre Syndrome
GD3: Disialyloganglioside
GM1: Monosialotetrahexosylganglioside
IVIG: Immune globulin for intravenous administration (also abbreviated IgIv or IGIV)
MAC: Membrane attack complex (of Complement, C5b-9)
MMN: Multifocal motor neuropathy
MOA: Mechanism of action
NMO: Neuromyelitis Optica, (NMO-spectrum disorder)
PD: Pharmacodynamics
PK: Pharmacokinetics
PLEX: Plasma exchange (Plasmapheresis)
RCT: Randomized controlled trial
SCIG: Subcutaneously administered IgG (IGSC)
SmPC: Summary of Product Characteristics (EMA)
